# Ideal Anatomical Landmark Points for Thoracic Esophagus Segmentation in the Chinese Population

**DOI:** 10.3389/fsurg.2021.729694

**Published:** 2021-12-14

**Authors:** Di Lu, Xiuyu Ji, Jintao Zhan, Jianxue Zhai, Tingxiao Fang, Siyang Feng, Xiguang Liu, Lin Yu, Zhiming Chen, Zhizhi Wang, Xuanzhen Wu, Sue Liu, Hua Wu, Kaican Cai

**Affiliations:** ^1^Department of Thoracic Surgery, Nanfang Hospital, Southern Medical University, Guangzhou, China; ^2^Department of Thoracic Surgery, Shunde Hospital of Southern Medical University, Foshan, China; ^3^School of Biomedical Engineering, Southern Medical University, Guangzhou, China

**Keywords:** esophagus, segmentation, anatomical landmark, coefficient of variation, thoracic surgery

## Abstract

**Introduction:** The standards of esophagus segmentation remain different between the Japan Esophageal Society (JES) guideline and the Union for International Cancer Control (UICC)/American Joint Committee on Cancer (AJCC) guideline. This study aimed to present variations in the location of intrathoracic esophageal adjacent anatomical landmarks (EAALs) and determine an appropriate method for segmenting the thoracic esophagus based on the relatively fixed EAALs.

**Patients and Methods:** The distances from the upper incisors to the upper border of the esophageal hiatus, lower border of the inferior pulmonary vein (LPV), tracheal bifurcation, lower border of the azygous vein (LAV), and thoracic inlet were measured in the patients undergoing thoracic surgery. The median distances between the EAALs and the specified starting points, as well as reference value ranges and ratios, were obtained. The variation coefficients of distances and ratios from certain starting points to different EAALs were calculated and compared to determine the relatively fixed landmarks.

**Results:** This study included 305 patients. The average distance from the upper incisors to the upper border of the cardia, the midpoint between the tracheal bifurcation and esophageal hiatus (MTBEH), LPV, LAV, tracheal bifurcation, and thoracic inlet were 41.6, 35.3, 34.8, 29.4, 29.5, and 20.3 cm, respectively. The distances from the upper incisors or thoracic inlet to any intrathoracic EAALs in men were higher than in women. In addition, the height, weight, and body mass index (BMI) were correlated with the distances. The ratio of the distance between the upper incisors and tracheal bifurcation to the distance between the upper incisors and upper border of the cardia and the ratio of the distance between the thoracic inlet and tracheal bifurcation to the distance between the thoracic inlet and upper border of the cardia possessed relatively smaller coefficients of variation.

**Conclusion:** The distances from the EAALs to the upper incisors vary with height, weight, BMI, and gender. Compared with distance, the ratios are more suitable for esophagus segmentation. Tracheal bifurcation and MTBEH are ideal EAALs for thoracic esophagus segmentation, and this is consistent with the JES guideline recommendation.

## Introduction

Esophageal cancer is one of the most common cancers worldwide, and due to its poor prognosis, is one of the most common causes of cancer-related mortality (1). The Japan Esophageal Society (JES) guidelines and the Union for International Cancer Control (UICC)/American Joint Committee on Cancer (AJCC) guidelines are the two most widely used standards worldwide for the diagnosis and treatment of esophageal cancer.

Harushi Udagawa and Masaki Ueno compared the latest eighth edition of UICC and 11th edition of JES. They pointed out that inadequate attention to tumor location might be one of the reasons leading to inferior efficiency of the UICC compared with the JES in describing the spread of a given esophageal cancer (2). It has been reported by many studies that the primary site of esophageal cancer is an independent prognostic factor (3–6), and it might be associated with the acute adverse events of subsequent chemoradiotherapy (7), as well as tumor recurrence (8, 9).

However, the differences exist between the two guidelines for the segmentation standards of the esophagus. According to the JES, the esophagus is divided into three main parts: cervical esophagus (this extends from the esophageal orifice to the sternal notch), thoracic esophagus (from the sternal notch to the superior margin of the esophageal hiatus), and abdominal esophagus (from the superior margin of the esophageal hiatus to the esophagogastric junction). The thoracic esophagus is further divided into three segments: the upper thoracic esophagus (from the sternal notch to the tracheal bifurcation), the middle thoracic esophagus (the proximal half of the two equal portions between the tracheal bifurcation and the esophagogastric junction), and the lower thoracic esophagus (the thoracic part of the distal half of the two equal portions between the tracheal bifurcation and the esophagogastric junction) (10). According to the UICC/AJCC guideline, the esophagus is also divided into three parts (the cervical, thoracic, and abdominal part). The cervical and abdominal esophagi are the same as those defined by the JES. According to the UICC/AJCC, the thoracic part is divided into three segments: the upper thoracic esophagus is bordered superiorly by the thoracic inlet and inferiorly by the lower border of the azygos vein; the middle esophagus is bordered superiorly by the lower border of the azygos vein and inferiorly by the inferior pulmonary veins; the lower thoracic esophagus is bordered superiorly by the inferior pulmonary veins and inferiorly by the stomach (11). Though both the AJCC and JES guidelines employ esophageal adjacent anatomical landmarks (EAALs) to segment the thoracic esophagus, none of them are based on objective data. In addition, the AJCC provides a reference range of the distances from the incisors to the anatomical landmarks for segmentation but points out that the reference may be affected by the height of the patient's. It has been reported that the length of the esophagus is related to height (12–15) and gender (16, 17). Thus, it could be inaccurate to use the distance from the incisors to the tumor to identify the tumor location during daily practice.

Furthermore, to the best of our knowledge, no studies have investigated esophagus segmentation in the Chinese patients. The Chinese, a population with the highest worldwide incidence rate of esophageal cancer as reported by the WHO in 2017, follow the esophagus segmentation standard by the AJCC. However, it is still debated whether this esophagus segmentation standard fits the Chinese population.

This study investigated how the esophageal adjacent anatomical landmarks are affected by the height, weight, gender, BMI, and age. In addition, relatively fixed EAALs to guide the anatomical segmentation of the thoracic esophagus were identified based on the objective data of the Chinese patients. For this, a novel measuring method called the “nasogastric tube fiber measurement method” was used to find the ideal EAALs for the segmentation of the thoracic esophagus in the Chinese population.

## Patients and Methods

### Measuring Methods

**Figure 1:**
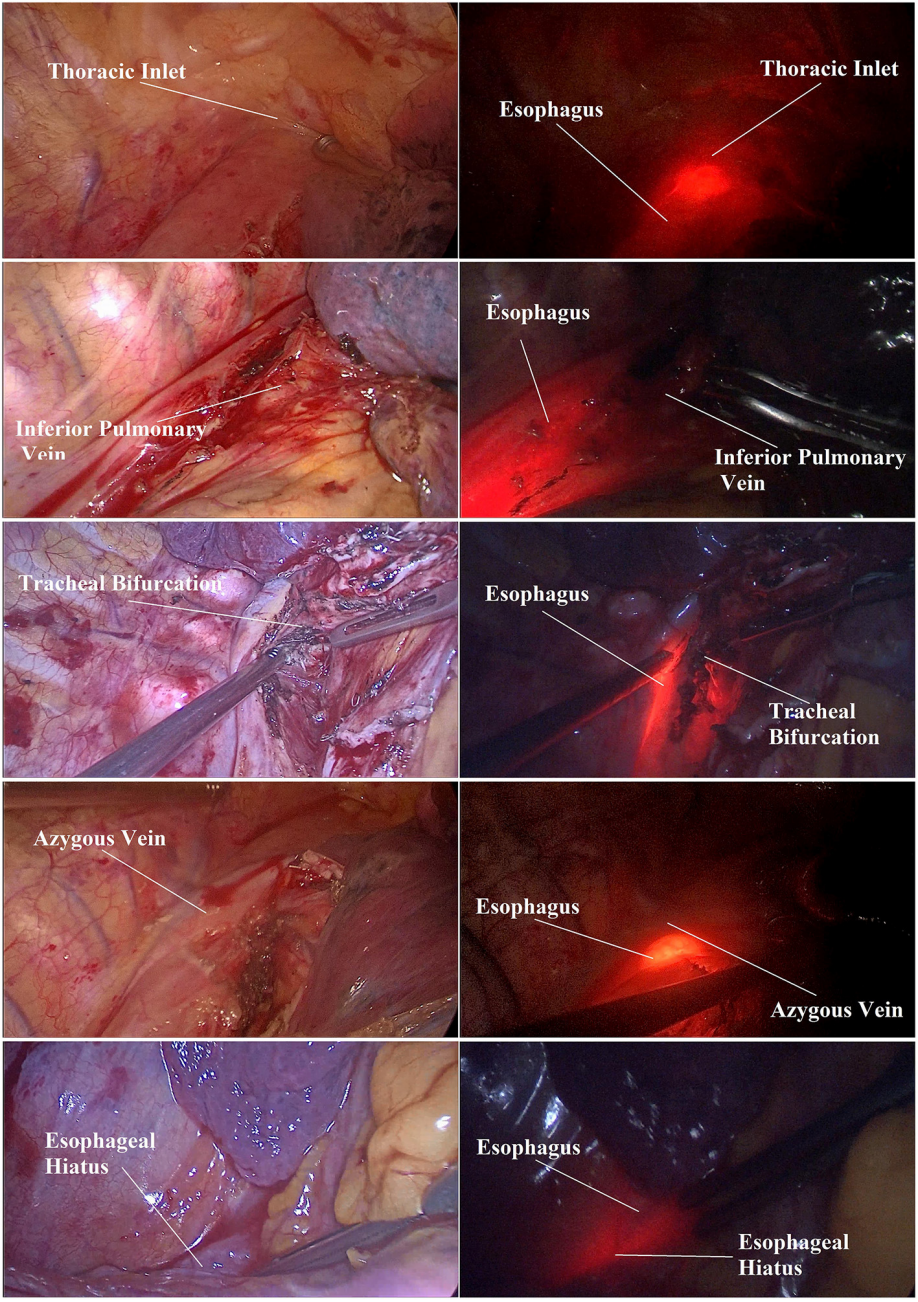
Real-time and intuitive measurement scene during surgery.

The “nasogastric tube fiber measurement method” was invented and adopted in this study. As shown in Figure 1, an 18F nasogastric tube(Safeed, Terumo Medical Products, Hangzhou, China) and optic fiber device (ZX-150L, Zhongxun Optics Instrument, Shenzhen, China) were customized to ensure that the nasogastric tube could accommodate the insertion of the optic fiber at any time during the surgery. The optic fiber was illuminated by a light source. Furthermore, the optic fiber could be seen through the esophageal wall when the light source was turned on. Before the surgery, the elaborated gastric tube was inserted 45 cm from the upper incisors into the digestive tract to ensure that its bottom entered the stomach. Next, the negative pressure drainage bottle was used to drain the gastric juice. Then, the nasogastric tube was inserted forward to 70 cm from the upper incisors, and the fiber was inserted to 45 cm from the upper incisors so that the tip of the fiber was far beyond the esophageal hiatus. In this way, the photothermal injuries from possible direct interaction between the fiber and esophageal tissue could be avoided to the greatest extent. With that, the light source was turned on to mark the anatomical structure at the upper border of the fiber optic ring (Figure 1), and the distance from the esophageal hiatus and thoracic inlet was recorded. Finally, the distance between the upper incisors and the edge of each landmark was measured and recorded. Each distance was measured three times. Every time the fiber reached each anatomical landmark during the measuring process, the light source was turned off to prevent the mucosa from the potential burning injuries. After all the measurements were completed, the nasogastric tube and the optical fiber were removed. The fiber was cleaned, packaged, and disinfected for next-time use.

The distances from the upper incisors to the selected EAALs were measured directly, and the ratio of them to the distance between the upper incisors and esophageal hiatus was calculated. Furthermore, the distance from the thoracic inlet to these EAALs and the ratios of them to the distance between the thoracic inlet and esophageal hiatus were also calculated using the measured data. For the thoracic surgeries on the right side, the distance from the upper incisors to the esophageal hiatus, inferior pulmonary vein (LPV), tracheal bifurcation, lower border of the azygous vein (LAV), and thoracic inlet could be measured. For the thoracic surgeries on the left side, the distance from the upper incisors to the esophageal hiatus, LPV, tracheal bifurcation, and thoracic inlet could be measured, but the distance to the LAV could not be measured. Due to the limitations of the surgical methods, the abdominal esophagus could not be explored, and the distance from the upper incisors to the esophagogastric junction could not be measured.

### Study Design and Participants

Data used in this study were obtained from the patients admitted from July 2018 to December 2019 to the Department of Thoracic Surgery, Nanfang Hospital, Southern Medical University, Guangdong Province, China. The demographic baseline data of the participants were recorded, such as height, weight, gender, age, location of the lesion, and surgical procedure. All the measurements were performed by one specific person. The study was approved and authorized by the Nanfang Hospital Ethics Committee of Southern Medical University (approval number NFEC-2018-85) and registered online (ClinicalTrials.gov Identifier: NCT03720405). An informed consent was obtained from all the patients.

The participants included in this study met the following conditions: not younger than 18 years of age but not older than 90 years; without cardiac, liver, kidney, or other organic disabilities so that the patients could tolerate general anesthesia; without blood system diseases or recent coagulopathy; without serious infectious diseases; without serious gastroesophageal primary lesions or gastroesophageal malformations; without serious liver diseases that might result in the gastroesophageal varices; without upper gastrointestinal bleeding due to unknown reasons; without severe thoracocyllosis, spinal deformities, or dysplasia; in need of surgery in the intrathoracic organs or adjacent organs; able to reveal the corresponding anatomical structures clearly during the surgery; without changes in the intraoperative condition that would make continuing measurements impossible; without difficulties in inserting the nasogastric tube; and without the patients needing tracheal intubation under general anesthesia that might adversely affect insertion of the gastric tube.

### Statistical Analysis

Using the descriptive statistics, the median distances between the EAALs and the specified starting points, as well as the reference value ranges of the distances, were obtained.

A two-way ANOVA analysis was performed to explore the differences in the distances between men and women. A linear regression was performed to find the relationship among the distances and height, weight, and body mass index (BMI). The value of *P* < 0.05 was considered statistically significant.

The original data were selected and divided into 16 groups depending on the four different EAALs (LPV, midpoint between tracheal bifurcation and esophageal hiatus (MTBEH), LAV, tracheal bifurcation) and the outcomes using four different kinds of calculating methods (distance from the upper incisors to EAALs, the ratios of distances between the upper incisors and selected EAALs to between the upper incisors and upper border of the cardia, distances from the thoracic inlet to the selected EAALs, the ratios of distances between the thoracic inlet and selected EAALs to between the thoracic inlet and upper border of the cardia). The results were converted into new data using the Box-Cox transformation equation to better obey normal distribution. Then, the converted data were put into the following formula to obtain the coefficient of variation (CV) for each group:


(1) $$\begin{array}{l} CV=Sd/mean\;*\;100\% \end{array}$$

where *mean* represents the mean value of each group, and *Sd* represents the SD of each group.

The significance test method was used to compare the coefficients of variation using the following formulas:


(2) $$\begin{array}{l} \alpha_{1}\;=\;\frac{\alpha }{k} \end{array}$$

(3) $$\begin{array}{l} u\;=\;\frac{{CV}_{1}-{CV}_{2}}{\sqrt{\frac{CV_{1}^{2}\left(1+2CV_{1}^{2}\right)}{2n_{1}}+\frac{CV_{2}^{2}\left(1+2CV_{2}^{2}\right)}{2n_{2}}}} \end{array}$$

In formula (2), α_1_ represents the CV significance test level, which was determined according to the Bonferroni method, where α = 0.05 and *k* is the number of groups to be compared. In formula (3), *CV*_1_ and *CV*_2_ represent the two coefficients of variation to be compared, and *n*_1_ and *n*_2_ are the sample numbers of the respective groups. When *u* > *t*_***α**n*1_ boundary value, *P* < α_1_ indicates that the difference is statistically significant. Otherwise, there is no statistical significance.

Finally, the relatively smaller CVs of the different EAALs calculated by the different methods were determined, which were consequently used to determine the relatively fixed landmarks.

The data were processed using SPSS 25.0 (IBM Corporation, Armonk, NY, USA) and R software 3.6.1 (R Foundation for Statistical Computing, Vienna, Austria).

## Results

### Baseline Characteristics

A total of 305 patients were finally included in this study. The mean age of the studied patients was 56 ± 1.33 years, the average height was 164.4 ± 0.89 cm, the average weight was 60.50 ± 1.19 kg, and the mean BMI was 22.3 ± 0.35 kg/m^2^. Among the 305 studied patients, 226 underwent lobectomy, 18 underwent pulmonary segmentectomy, and 21 underwent other surgical procedures, such as wedge resection, double lobectomy, pneumonectomy, and sleeve resection (Table 1).

**Table 1:** Demographic data (*N* = 305).

**Characteristic**	**No**.	**Value**
Age (years)	–	56 ± 1.33
Sex		
Male	195	–
Female	110	–
Height (cm)	–	164.4 ± 0.89
Weight (kg)	–	60.50 ± 1.19
BMI (kg/m^2^)	–	22.3 ± 0.35
Operative procedure		
Radical lobectomy	266	–
Radical segmentectomy	18	–
Other surgery	21	–

*Values are presented as the mean ± SD. BMI, body-mass index*.

### Distances and Ratios

As shown in Table 2, the median distances from the upper incisors to the esophageal hiatus, MTBEH, LPV, LAV, tracheal bifurcation, and thoracic inlet were 41.6, 35.3, 34.8, 29.4, 29.5, and 20.3 cm, respectively. The median distances from the thoracic inlet to the esophageal hiatus, MTBEH, LPV, LAV, and tracheal bifurcation were 21.0, 15.1, 14.1, 8.1, and 9.1 cm, respectively.

**Table 2:** Observations of the lengths and ratios of esophageal adjacent anatomical landmarks (EAALs) to the incisors/thoracic inlet.

**Landmark point**	**Male**		**Female**		**All**	
	**Length (quartile), cm**	**Ratio (quartile)**	**Length (quartile), cm**	**Ratio (quartile)**	**Length (quartile), cm**	**Ratio (quartile)**
Esophageal hiatus—incisors	42.6(40.0–45.1)	1	40.2(36.9–42)	1	41.6(37.8–44.1)	1
MTBEH—incisors	36.3(33.7–38.2)	0.86(0.84–0.88)	33.7(31.4–35.6)	0.85(0.83–0.87)	35.3(32.4–37.5)	0.86(0.84–0.87)
LPV—incisors	35.5 (33.5–37.8)	0.83(0.8–0.87)	32.6(31.0–35.0)	0.84(0.80–0.87)	34.8(31.9–37.3)	0.83(0.80–0.87)
LAV—incisors	30.0(27.3–32.0)	0.70(0.67–0.74)	27.8(24.0–30.4)	0.69(0.65–0.73)	29.4(26.2–31.8)	0.70(0.65–0.74)
Tracheal bifurcation—incisors	30.2(28.1–32.7)	0.72(0.68–0.75)	28.1(25.8–30.0)	0.70(0.66–0.74)	29.5(27.5–32.0)	0.71(0.67–0.75)
Thoracic inlet—incisors	21.1(18.92–23.3)	0.49(0.45–0.53)	19.5(16.9–21.9)	0.49(0.43–0.54)	20.3(18.3–23.0)	0.49(0.44–0.53)
Esophageal hiatus—thoracic inlet	21.8(19.5–24.0)	1	20.2(18.7–22.2)	1	21.0(19.1–23.2)	1
MTBEH—thoracic inlet	15.3(13.9–17.2)	0.72(0.69–0.75)	14.0(12.7–15.6)	0.71(0.68–0.74)	15.1(13.4–16.7)	0.72(0.68–0.75)
LPV—thoracic inlet	14.7(12.9–16.7)	0.67(0.6–0.7)	13.5(11.6–15.0)	0.65(0.69–0.72)	14.1(12.5–16.0)	0.66(0.61–0.73)
LAV to thoracic inlet	8.6(7.1–10.0)	0.40(0.33–0.50)	7.6(6.6–9.3)	0.38(0.31–0.45)	8.1(7.0–9.8)	0.39(0.33–0.46)
Tracheal bifurcation to thoracic inlet	9.4(8.0–12.3)	0.47(0.40–0.54)	8.8(7.3–10.1)	0.38(0.30–0.43)	9.1(7.5–11.0)	0.43(0.37–0.50)

*MTBEH: midpoint between the tracheal bifurcation and the esophagogastric junction*.

Taking the distances from the upper incisors to the esophageal hiatus as a controlled overall distance, the median ratios of the distances from the upper incisors to MTBEH, LPV, LAV, tracheal bifurcation, and thoracic inlet were 0.85, 0.84, 0.71, 0.71, and 0.49, respectively. Compared with the distance from the thoracic inlet to the esophageal hiatus, which was taken as a controlled overall distance, the median ratios of the distances from the upper incisors to MTBEH, LPV, LAV, and tracheal bifurcation were 0.72, 0.67, 0.39, and 0.43, respectively.

### Factors Influencing the Distances

**Figure 2:**
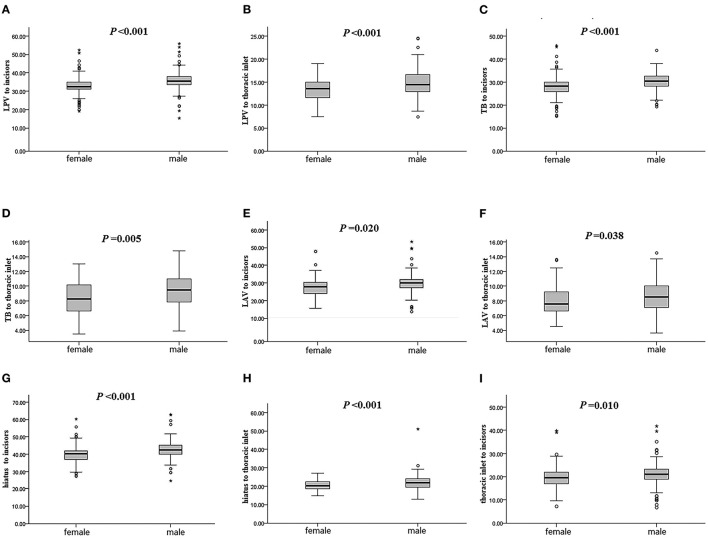
Length differences between males and females. Differences between males and females in distance from the incisors to the lower border of the inferior pulmonary vein **(A)**, from the thoracic inlet to the lower border of the inferior pulmonary vein **(B)**, from the incisors to the tracheal bifurcation **(C)**, from the thoracic inlet to the tracheal bifurcation **(D)**, from the incisors to the lower border of the azygous vein **(E)**, from the thoracic inlet to the lower border of the azygous vein **(F)**, from the incisors to the superior edge of the cardia **(G)**, from the thoracic inlet to the superior edge of the cardia **(H)**, and from the incisors to the thoracic inlet **(I)**. LPV, lower border of the inferior pulmonary vein; TB, tracheal bifurcation; LAV, lower border of the azygous vein. *difference is statistically significant.

The distance from the upper incisors to any EAAL in men was higher than in women, as was the distance from the thoracic inlet to any EAAL (Figure 2).

The heights of the patients correlated with the distances from the upper incisors to the esophageal hiatus, LPV, tracheal bifurcation, and thoracic inlet but not LAV. Similarly, the heights of the patients correlated with the distances from the thoracic inlet to the esophageal hiatus, LPV, and tracheal bifurcation but not LAV.

The weights of the patients correlated with the distances from the upper incisors to the esophageal hiatus, LPV, tracheal bifurcation, and thoracic inlet but not LAV. However, they did not correlate with the distances from the thoracic inlet to the esophageal hiatus, LPV, tracheal bifurcation, or LAV (Table 3).

**Table 3:** A descriptive correlation analysis between the distance from the incisors to certain EAALs and height, weight, or body-mass index (BMI).

**Landmark**	**The incisor group**			**The inlet group**		
	**Height**	**Weight**	**BMI**	**Height**	**Weight**	**BMI**
Esophageal hiatus	*r* = 0.247, *P* < 0.01	*r* = 0.155, *P* < 0.05	*P* > 0.05	*r* = 0.184 *P* < 0.01	*P* > 0.05	*r* = -0.192, *P* < 0.01
LPV	*r* = 0.235 *P* < 0.01	*r* = 0.168, *P* < 0.01	*P* > 0.05	*r* = 0.201, *P* < 0.01	*P* > 0.05	*r* = -0.196, *P* < 0.01
LAV	*P* > 0.05	*r* = 0.189, *P* > 0.01	*P* > 0.05	*P* > 0.05	*P* > 0.05	*P* > 0.05
Tracheal bifurcation	*r* = 0.231 *P* < 0.01	*r* = 0.187 *P* < 0.01	*P* > 0.05	*r* = 0.140 *P* < 0.05	*P* > 0.05	*P* > 0.05
Thoracic inlet	*r* = 0.171, *P* < 0.05	*r* = 0.251, *P* < 0.01	*r* = 0.174, *P* < 0.01	–	–	–

*BMI, body-mass index. r, correlation coefficient*.

The BMIs of the patients were not correlated with the distances from the upper incisors to the esophageal hiatus, LPV, LAV, or tracheal bifurcation but weakly correlated with the distance from the upper incisors to the thoracic inlet. They were also negatively correlated with the distance from the thoracic inlet to the esophageal hiatus and LPV but not LAV or tracheal bifurcation (Table 3).

## Variability of the Ratios and Distances

As several factors could influence the distance from the EAALs to the upper incisors, further studies were performed to explore the relatively fixed EAALs.

### Naming the CVs

**Figure 3:**
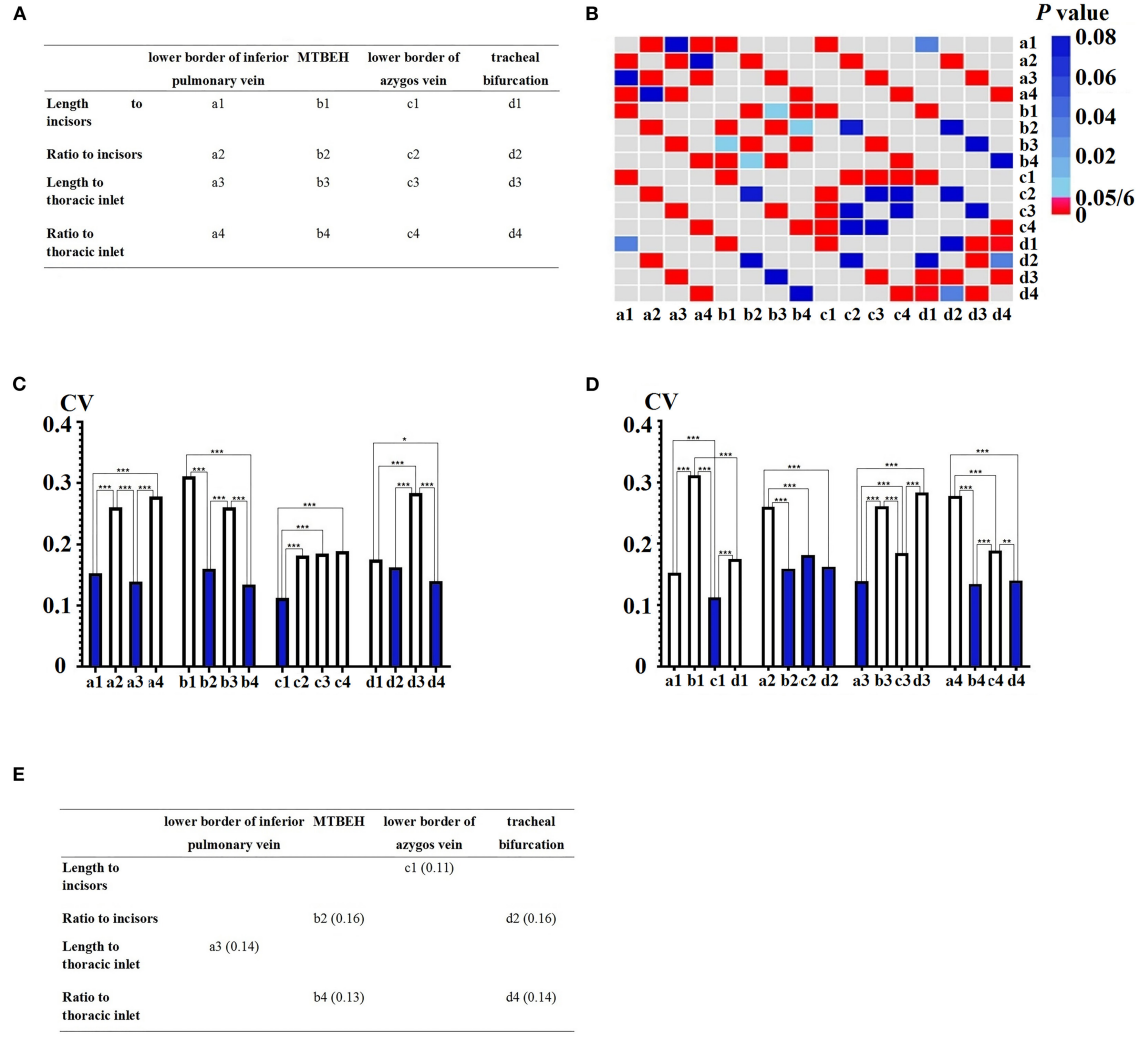
Variability of the ratios and lengths. **(A)** Naming the coefficient of variations (CVs) of the different esophageal adjacent anatomical landmarks (EAALs) measured by the different methods. **(B)** The *p* values between different CVs. **(C)** CV difference in the groups of different EAALs. **(D)** CV difference in the groups of different measuring methods. **(E)** The relatively smaller CVs not only in the respective measurement method groups but also in the respective EAAL groups. CV, coefficient of variation. MTBEH: midpoint between tracheal bifurcation and esophageal hiatus. *difference is statistically significant.

As shown in Figure 3A, the CVs of the different EAALs calculated by different methods were named respectively from a1 to d4. For example, the CV of the distance from the LPV to the upper incisors was labeled as a1, while the CV of the ratio of the distance between the LPV and upper incisors to between the upper incisors and upper border of the cardia was labeled as a2. Similarly, the CV of the distance from the LPV to the thoracic inlet was labeled as a3, while the CV of the ratio of the distance between the LPV and thoracic inlet to between the thoracic inlet and upper border of the cardia was labeled as a4. The other CVs were renamed in the same manner.

### The Relatively Smaller CVs When Grouped by Different EAALs

When grouped by different EAALs, a3 was smaller than a2 and a4 (0.14 vs. 0.26, *P* < 0.05/6; 0.14 vs. 0.28, *P* < 0.05/6) in the LPV group but was not significantly different from a1 (0.14 vs. 0.15, *P* ≥ 0.05/6) (Figures 3B,C).

Similarly, b2 and b4 were smaller than b1 and b3 in the MTBEH group, while c1 was smaller than c2, c3, and c4 in the LAV group. In addition, d2 and d4 were smaller than d1 and d3 in the tracheal bifurcation group (Figures 3B,C).

### The Relatively Smaller CVs When Grouped by Different Calculation Methods

Grouped by different EAALs, c1 was smaller than a1, b1, and d1 (0.11 vs. 0.15, *P* < 0.05/6; 0.11 vs. 0.31, *P* < 0.05/6; 0.11 vs. 0.17, *P* < 0.05/6) in distance to the upper incisors group (Figures 3B,D).

Similarly, b2, c2, and d2 were smaller than b1 in ratio to the upper incisors group, while a3 was smaller than b3, c3, and d3 in distance to the thoracic inlet group. Additionally, b4 and d4 were smaller than a4 and c4 in ratio to the thoracic inlet group (Figures 3B,D).

### The Relatively Fixed EAALs Calculated by Different Methods

According to the results from Sections Naming the CVs and The Relatively Smaller CVs When Grouped by Different EAALs, a1, a3, b2, b4, c1, d2, and d4 were relatively smaller CVs in the respective calculating method groups, while c1, b2, c2, d2, a3, b4, and d4 were relatively smaller CVs in the respective EAALs groups. Among these CVs, a3, b2, b4, c1, d2, and d4 were relatively smaller CVs not only in the respective calculating method groups but also in the respective EAALs groups (Figure 3E). For this reason, they were considered to be more fixed than a1 and c2.

Among the six CVs, one (c1) supported that distance to the upper incisors was a relatively fixed calculating method, two (b2 and d2) supported the ratio to incisors, one (a3) supported the distance to the thoracic inlet, and two (b4 and d4) supported the ratio to the thoracic inlet. In other words, one (a3) supported LPV as a relatively fixed EAAL, two (b2 and b4) supported MTBEH, one (c1) supported LAV, and two (d2 and d4) supported tracheal bifurcation. Therefore, the ratio to incisors and ratio to thoracic inlet were selected as relatively fixed calculating methods, while MTBEH and tracheal bifurcation were selected as relatively fixed EAALs.

Thus, b2, b4, d2, and d4 were considered relatively smaller CVs than a3 and c1. Further comparison by the specific value of these four CVs, b4 (0.13) and d4 (0.14) were smaller than b2 (0.16) and d2 (0.16) (Figure 3E). Combining all the results above, b4 and d4 were finally selected as the relatively smaller CVs among all the CVs, indicating that the ratio of MTBEH to the thoracic inlet and that of tracheal bifurcation to thoracic inlet were relatively fixed among the different EAALs calculated by the different methods.

## Discussion

In this study, by collecting the distances from selected EAALs to the upper incisors and thoracic inlet, and calculating their related ratios, we analyzed the median values of the distances and ratios, the factors influencing the lengths, as well as the relatively fixed calculating methods and EAALs. Based on the data of the Chinese patients, the findings of this study may be more suited to the Chinese patients with esophageal cancer. However, it could be used as a reference for other countries and races.

Tae Jin Song reported that the mean distances from the upper incisors to the cricopharyngeal narrowing, aortic arch, left main bronchus, hiatus, and esophagogastric junction were 16.43 ± 0.80, 26.29 ± 2.14, 29.06 ± 2.51, 39.93 ± 2.53, and 42.62 ± 2.42 cm, respectively (14). The mean distance of esophagus of an adult was 28.3 ± 2.41 cm as reported by Ziad T et al. (13) and 23.42 ± 2.02 cm as reported by Zengye Wang et al. (17). Xiaohong Wei and partners reported that the average distance from the upper incisors to the cardia was 44.4 cm, and from the upper end of the esophagus to the cardia was 28.0 cm (12). In this study, the median distance from the upper incisors to the esophageal hiatus was 41.6 cm, while the median distances from the upper incisors to the LPV, LAV, tracheal bifurcation, and thoracic inlet were 34.8, 29.4, 29.5, and 20.3 cm, respectively. The difference in the distances related to the esophagus reported in the different articles might result from the different nationalities or races of the patients studied.

The height, distance from the seventh cervical vertebra to the coccyx, and the distance from the upper incisors to the occiput were reported by Tae Jin Song to significantly correlate with the true esophageal lengths (14). Ziad et al. reported a trend for longer esophagus lengths in men, but gender was no longer related to the length after adjusting for height (13). Additionally, Xiaohong Wei reported a positive correlation between the length of the esophagus and body height in 91.3% of their studied cases (12). Conversely, Zengye Wang reported that the length of the esophagus was longer in men than in women but showed no significant relation with height (17), which was similar to the results reported by Li Qing (16). It was indicated from this study that the distances from the EAALs to the upper incisors in men were larger than in women. The height, weight, and BMI were, to some extent, correlated with the distances from the EAALs to the upper incisors or thoracic inlet. However, the correlation coefficients were calculated to be low, indicating that the other factors might influence the esophageal length. Sufficient explanatory factors are not found yet to predict the distance from each EAAL to the upper incisors or thoracic inlet. This requires further research to find the appropriate and relevant factors to establish a complete and accurate multiple regression equation.

The EAALs are not easily affected by self-variation, and secondary factors may be more conformant with the requirements for thoracic esophageal segmentation. The azygous venous arch has obvious positional changes due to compression or invasion from the esophageal tracheal mass, surrounding lymph nodes, and changed superior vena cava, which may cause deviations when segmenting the esophageal tumor (18, 19). In contrast, few variations occurred in the carina, and the displacements were not obvious when the tumors invaded it, though the cases of pseudo-protuberance and bilateral tracheal and bronchial variations occasionally emerged (20, 21). Some literature has reported variations in the lower pulmonary vein, which is complex and with a high mutation rate (22). MTBEH is situated on the esophagus and has few physiological variations. Therefore, it could be used in the segmentation of the middle and lower thoracic esophagus. In this study, by comparing the CVs of different EAALs calculated by different methods, it was found that the ratio of MTBEH to thoracic inlet and ratio of tracheal bifurcation to thoracic inlet were relatively fixed, making them suitable for the thoracic esophageal segmentation.

In this study, the esophageal hiatus was used in the thoracic esophagus segmentation instead of the esophagogastric junction due to the following reasons: (1) the main aim of this study was to explore a better segmentation standard for the thoracic esophagus rather than the abdominal esophagus or other portions of the esophagus. (2) There remains a discrepancy in the accurate allocation of the cardia (23). This may result in the difficulties in finding the cardia and errors in measuring the results. (3) The distance from the upper incisors to the esophagogastric junction was not fixed (42.61 ± 2.42 cm) (14). (4) The abdominal esophagus is contained in the lower thoracic esophagus by the AJCC but not by the JES. However, most tumors located in the abdominal esophagus can be categorized as cancers of the esophagogastric junction. An agreement on their definition and treatment, such as the choice of surgical procedure, is not yet reached (6, 11, 24). (5) It was unfeasible to expose the abdominal esophagus, the esophagogastric junction, and the cardia during the intrathoracic surgeries.

There were some intrinsic limitations in this study. First, the surgeons' determinations of different EAALs on the fiber were not always consistent, causing the subjective measurement errors. Second, the operation of measurement was relatively invasive, which required strict controls of the measurement procedures to avoid the esophageal mucosal damage. Third, all the patients included in this study were the Chinese patients who were diagnosed at the Nanfang Hospital. Therefore, it should be further studied whether the results can be adapted to the patients of different nationalities or races. Fourth, other methods for measuring the length of the esophagus besides our “nasogastric tube fiber measurement method,” such as ultrasonic imaging and CT imaging, are available. Nevertheless, which method is superior remains unknown. Fifth, the results of this study were based on the patients without esophageal tumors. As the influence of esophageal tumors on the esophagus length is seldomly studied, it remains unknown whether the results of this study would be the same as the results from the patients suffering from the esophageal tumors. Sixth, we used a method to cross-compare the CVs in different groups to select the relatively fixed EAALs rather than directly comparing all the CV values. This resulted in the values of some other CVs being smaller than the values of the CVs we selected. Thus, it is worthy of further discussion whether the former method is better. However, it was suggested not to compare the CVs directly in the multi-group data.

In conclusion, what we report here is that (1) the distances from the EAALs to the upper incisors vary with the height, weight, BMI, and gender of the patient, and that the ratios are more suitable than the lengths for thoracic esophagus segmentation; (2) the landmarks of tracheal bifurcation and MTBEH are relatively ideal EAALs for the thoracic esophagus segmentation, a result that is consistent with the JES guideline recommendation.

## Data Availability Statement

The original contributions presented in the study are included in the article/Supplementary Material, further inquiries can be directed to the corresponding author/s.

## Ethics Statement

The studies involving human participants were reviewed and approved by Nanfang Hospital Ethics Committee of Southern Medical University. The patients/participants provided their written informed consent to participate in this study.

## Author Contributions

DL, XJ, and KC conceptualized the study. DL, JZhan, XJ, and JZhai designed the study. XJ performed the measurements. JZhai, XJ, SL, TF, and XL acquired the data. DL, JZhan, JZhai, LY, and SF analyzed the data. ZW, ZC, and XW drew the statistical figures and tables. DL and XJ wrote the first draft of the manuscript. HW and KC revised the manuscript. All authors reviewed the manuscript and approved the final version of the manuscript.

## Conflict of Interest

The authors declare that the research was conducted in the absence of any commercial or financial relationships that could be construed as a potential conflict of interest.

## Publisher's Note

All claims expressed in this article are solely those of the authors and do not necessarily represent those of their affiliated organizations, or those of the publisher, the editors and the reviewers. Any product that may be evaluated in this article, or claim that may be made by its manufacturer, is not guaranteed or endorsed by the publisher.

## Abbreviations

EAALs: esophageal adjacent anatomical landmarks
LPV: lower border of the inferior pulmonary vein
LAV: lower border of the azygous vein
MTBEH: midpoint between tracheal bifurcation and esophageal hiatus.

## References

[1] Lu D Liu X Li M Feng S Dong X Yu X. Three-port mediastino-laparoscopic esophagectomy (TPMLE) for an 81-year-old female with early-staged esophageal cancer: a case report of combining single-port mediastinoscopic esophagectomy and reduced port laparoscopic surgery. J Thorac Dis. (2018) 10:E378–82. 10.21037/jtd.2018.05.52 29997998 PMC6006129

[2] Udagawa H Ueno M. Comparison of two major staging systems of esophageal cancer-toward more practical common scale for tumor staging. Ann Transl Med. (2018) 6:76. 10.21037/atm.2018.01.27 29666799 PMC5890035

[3] Xu Q Liu ZK Cao YK Li YM Zhu SC. Relationship of gross tumor volume with lymph node metastasis and prognosis of esophageal carcinoma. Zhonghua Zhong Liu Za Zhi. (2012) 34:684–7. 10.3760/cma.j.issn.0253-3766.2012.09.009 23159082

[4] Wang Y Wang L Yang Q Li J Qi Z He M. Factors on prognosis in patients of stage pT3N0M0 thoracic esophageal squamous cell carcinoma after two-field esophagectomy. J Cancer Res Ther. (2015) 11 Suppl 1:C16–23. 10.4103/0973-1482.163833 26323918

[5] Yu Z Yang J Gao L Huang Q Zi H Li X. Competing risk analysis study of prognosis in patients with Esophageal Carcinoma 2006–2015 using data from the surveillance, epidemiology, and end results (SEER) database. Med Sci Monit. (2020) 26:e918686. 10.12659/MSM.918686 31966000 PMC6996264

[6] Rice TW Gress DM Patil DT Hofstetter WL Kelsen DP Blackstone EH. Cancer of the esophagus and esophagogastric junction-Major changes in the American Joint Committee on Cancer eighth edition cancer staging manual. CA Cancer J Clin. (2017) 67:304–17. 10.3322/caac.21399 28556024

[7] Hironaka S Komori A Machida R Ito Y Takeuchi H Ogawa G. The association of primary tumor site with acute adverse event and efficacy of definitive chemoradiotherapy for cStage II/III esophageal cancer: an exploratory analysis of JCOG0909. Esophagus. (2020) 17:417–74. 10.1007/s10388-020-00741-w 32342253

[8] Doki Y Ishikawa O Takachi K Miyashiro I Sasaki Y Ohigashi H. Association of the primary tumor location with the site of tumor recurrence after curative resection of thoracic esophageal carcinoma. World J Surg. (2005) 29:700–7. 10.1007/s00268-005-7596-4 16078126

[9] Ichikawa H Kosugi SI Kanda T Ishikawa T Yajima K Akazawa K. Prognostic significance of initial recurrence site in hematogenous recurrence of esophageal squamous cell carcinoma. Hepatogastroenterology. (2014) 61:2241–6. 25699360

[10] Japan Esophageal S. Japanese Classification of esophageal cancer, 11th edition: part I. Esophagus. (2017) 14:1–36. 10.1007/s10388-016-0551-7 28111535 PMC5222932

[11] Rice TW Ishwaran H Ferguson MK Blackstone EH Goldstraw P. Cancer of the esophagus and esophagogastric junction: an eighth edition staging primer. J Thorac Oncol. (2017) 12:36–42. 10.1016/j.jtho.2016.10.016 27810391 PMC5591443

[12] Wei XH. Measurement of the length of the adult esophagus using a fiberogastroscope: 104 cases. Zhonghua Wai Ke Za Zhi. (1989) 27:407–8. 2598742

[13] Awad ZT Watson P Filipi CJ Marsh RE Tomonaga T Shiino Y. Correlations between esophageal diseases and manometric length: a study of 617 patients. J Gastrointest Surg. (1999) 3:483–8. 10.1016/S1091-255X(99)80101-2 10482704

[14] Song TJ Kim YH Ryu HS Hyun JH. Correlation of esophageal lengths with measurable external parameters. Korean J Intern Med. (1991) 6:16–20. 10.3904/kjim.1991.6.1.16 1742251 PMC4535018

[15] Putnam PE Orenstein SR. Determining esophageal length from crown-rump length. J Pediatr Gastroenterol Nutr. (1991) 13:354–9. 10.1097/00005176-199111000-00004 1779308

[16] Li Q Castell JA Castell DO. Manometric determination of esophageal length. Am J Gastroenterol. (1994) 89:722–5. 8172145

[17] Wang ZY. The length of the esophagus measured by SND-1 esophagus detector. report of 197 cases. Zhonghua Wai Ke Za Zhi. (1991) 29:566–90. 1813262

[18] Arslan G Çubuk M Özkaynak C Sindel T Lüleci E. Absence of the azygos vein. Clin Imaging. (2000) 24:157–8. 10.1016/S0899-7071(00)00191-1 11150683

[19] Smathers RL Buschi AJ Pope TL Jr Brenbridge AN Williamson BR. The azygous arch: normal and pathologic CT appearance. AJR Am J Roentgenol. (1982) 139:477–83. 10.2214/ajr.139.3.477 6981309

[20] Doolittle AM Mair EA. Tracheal bronchus: classification, endoscopic analysis, and airway management. Otolaryngol Head Neck Surg. (2002) 126:240–3. 10.1067/mhn.2002.122703 11956531

[21] Rahmanian R Zheng J Chadha NK Kozak FK Campbell AI Ludemann JP. False carina: a distinct variant of tracheal bronchus. Int J Pediatr Otorhinolaryngol. (2015) 79:623–8. 10.1016/j.ijporl.2015.01.023 25683591

[22] Mansour M Holmvang G Sosnovik D Migrino R Abbara S Ruskin J. Assessment of pulmonary vein anatomic variability by magnetic resonance imaging: implications for catheter ablation techniques for atrial fibrillation. J Cardiovasc Electrophysiol. (2004) 15:387–93. 10.1046/j.1540-8167.2004.03515.x 15089984

[23] Lenglinger J See SF Beller L Cosentini E Asari R Wrba F. The cardia: esophageal or gastric? critical reviewing the anatomy and histopathology of the esophagogastric junction. Acta Chir Iugosl. (2012) 59:15–26. 10.2298/ACI1203015L 23654002

[24] Kumamoto T Kurahashi Y Niwa H Nakanishi Y Okumura K Ozawa R. True esophagogastric junction adenocarcinoma: background of its definition and current surgical trends. Surg Today. (2019) 50:809–14. 10.1007/s00595-019-01843-4 31278583

